# Molecular Structure Modulated Trap Distribution and Carrier Migration in Fluorinated Epoxy Resin

**DOI:** 10.3390/molecules25133071

**Published:** 2020-07-06

**Authors:** Jin Li, Yufan Wang, Zhaoyu Ran, Hang Yao, Boxue Du, Tatsuo Takada

**Affiliations:** 1 Key Laboratory of Smart Grid of Education Ministry, School of Electrical and Information Engineering, Tianjin University, Tianjin 300072, China; lijin@tju.edu.cn (J.L.); 15122557071@163.com (Y.W.); 2 Measurement and Electric Machine Control Laboratory, Tokyo City University, 1-28-1Tamazutsumi, Setagaya-ku, Tokyo 158-8557, Japan; takada@a03.itscom.net

**Keywords:** epoxy resin, surface charge, molecular structure, trap distribution, quantum chemical calculation

## Abstract

Surface charge accumulation on epoxy insulators is one of the most serious problems threatening the operation safety of the direct current gas-insulated transmission line (GIL), and can be efficiently inhibited by the surface modification technology. This paper investigated the mechanisms of fluorination modulated surface charge behaviors of epoxy resin through quantum chemical calculation (QCC) analysis of the molecular structure. The results show that after fluorination, the surface charge dissipation process of the epoxy sample is accelerated by the introduced shallow trap sites, which is further clarified by the carrier mobility model. The electron distribution probability of the highest occupied molecular orbitals (HOMO) under positive charging and the lowest unoccupied molecular orbitals (LUMO) under negative charging shows distinctive patterns. It is illustrated that electrons are likely to aggregate locally around benzenes for the positively charged molecular structure, while electrons tend to distribute all along the epoxy chain under negatively charging. The calculated results verify that fluorination can modulate surface charge behaviors of epoxy resin through redesigning its molecular structure, trap distribution and charging patterns.

## 1. Introduction

With the development of high-voltage direct current (HVDC) transmission, the gas-insulated transmission line (GIL) has been put into application all around the world [1,2,3]. Under the unipolar electric field, charges tend to deposit on the insulator surface during the long-term operation of the GIL, thus making the gas/solid interface the weak part of the whole system [4,5,6,7]. The local electric field concentration will be enhanced and may cause partial discharge, moreover, even results in flashover failure along the insulator surface [8,9]. As one of the main concerning topics, surface characteristics of the insulators are in urgent need to be improved, in which the structures of the epoxy resin play an extremely important role [10,11,12,13].

There exists a close relationship between the molecular structure and the macro properties of the epoxy resin. As a mature process, surface fluorination technology has been proved to be efficient for the modification of polymer materials in various researches, showing priorities of the simple operation and the low cost [14,15,16]. Studies have shown that in the fluorination process, F atoms can be introduced into the material and polymer chain breaking and crosslinking reactions may occur. The physical and chemical characteristics of the formed fluorinated surface layer depend on the fluorination conditions and the polymer material itself [17,18,19]. In addition, the regulation of the charge transport process can be attributed to the change of the surface state. Usually, charge migration has a great relation with the energy level distribution determined by molecular structure and its interaction [20,21].

Over the past few years, researchers have gradually focused on the molecular simulation and the quantum chemical calculation (QCC) of small size molecules to reveal the charge transport characteristics which greatly facilitates the researches of developing novel DC insulation materials and analyzing various space and surface charge behaviors [22,23,24,25]. The studies have attracted much concern on the physiochemical characteristics of the polymer by calculation and analysis from the molecule level [26,27,28]. The common theory is quantum theory, and quantum chemical calculation is used to show the structure of the molecules and the interaction between the molecules, helping to predict the electron behavior and figure out the material properties and their internal relationship with the structure. With the development of the framework and numerical methods, the density functional theory (DFT) has become a powerful research tool, for exploring the structural and the transport properties of the polymer material considering the effects of different electric fields [29,30,31,32,33]. However, the physical mechanism of fluorination modulated surface charge behaviors hasn’t been revealed from the point of molecular orbital view.

In this paper, surface charge behaviors of original and fluorinated epoxy samples were investigated through the surface potential decay (SPD) experiment. The trap distribution was calculated and compared. Then the quantum calculation model was established, based on which the trap distribution and molecular orbitals were obtained for positively and negatively charged epoxy, respectively. The relationship between the carrier migration and trap sites was also analyzed. The mechanism was discussed, providing a reference for the in-depth understanding of the physiochemical process during the fluorination treatment.

## 2. Results

### 2.1. Surface Charge Behaviors

**Figure 1:**
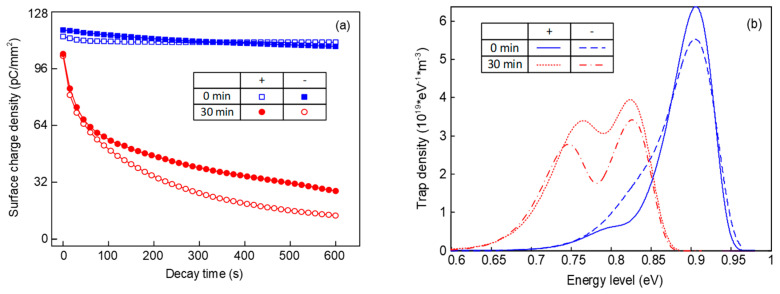
Surface potential decay process and trap distribution of epoxy resin before and after fluorination. (**a**) Relationship between the decay time and the surface charge density; (**b**) relationship between the energy level and the trap density of epoxy samples.

Figure 1 shows the surface charge decay process and trap distribution of epoxy before and after fluorination. It can be seen in Figure 1a that the surface charge dissipation rate is quite slow for the original epoxy samples, whether the polarity of the pre-corona charging voltage is positive or negative. After being treated by the surface fluorination for 30 min, the initial density of the surface charge is decreased significantly. In addition, the density value can be reduced to a half in approximately 200 s, with a much faster rate than that of the original one. Additionally, compared with the positive surface charges, the negative charges possess a higher dissipation speed, which applies to all the samples.

According to Figure 1b, original samples mainly contain deep traps under positive voltage. As for the trap distribution under negative voltage, there exist a few shallow traps and the density of the deep traps is decreased. After the fluorination, shallow traps of the sample are generated at about 0.73–0.78 eV, meanwhile the deep trap level decreases to 0.82–0.85 eV. There will inevitably be traps in the epoxy resin matrix due to the unsaturated bonds and branched chain structures, most of which are deep ones. When treated by the F_2_, the substitution and addition reactions also inevitably cause structural changes. These structural changes or disturbances caused by chain scission and substitution and addition reactions are equivalent to physical traps, which are known to possess a much shallower trap depth than that of chemical traps. Furthermore, the density of both the deep and shallow traps under negative voltage is lower than that under positive voltage, which will be discussed later.

### 2.2. Trap Site Modulation

**Figure 2:**
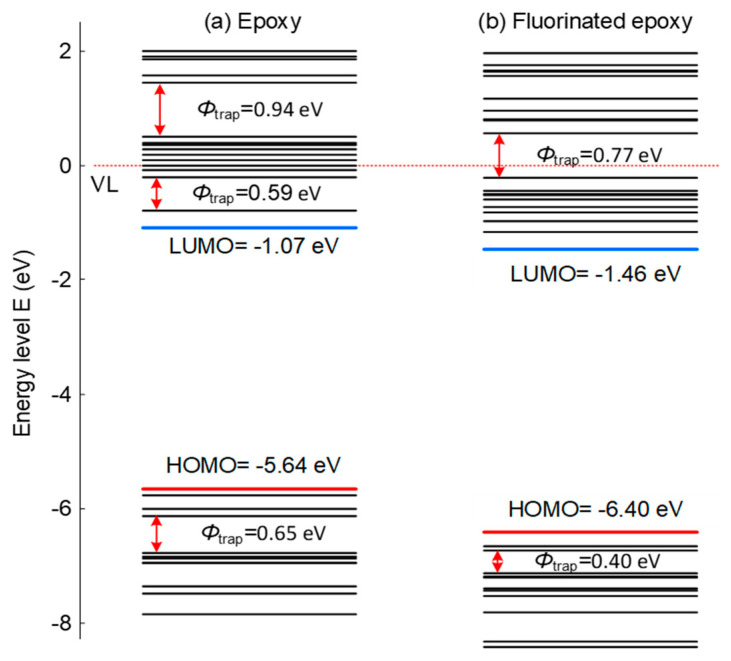
Energy level distribution and trap sites of epoxy resin before and after fluorination treatment.

Figure 2 shows the energy level and trap sites distribution of epoxy resin before and after fluorination treatment. For original epoxy, there are some apparent deep electron and hole traps, and correspondingly, surface charge dissipation is much slow. After fluorination, some of the deep electron trap sites disappear and some deep hole trap sites get much shallower. It should be noted that although the detailed trap sites should be obtained by locating the energy level with related molecular structure, such apparent trap sites can also reflect the changes before and after fluorination. Additionally, the increase of the band gap indicates the effects of the treatment, elevating the energy required by the electrons and raising the barrier of electron migration between different lattices.

## 3. Discussion

### 3.1. Carrier Hopping Process

The carrier migration velocity and hopping probability are calculated using Formulas (1) and (2).

(1) $$v=\mu E=\mu_{0}E\;\theta_{hop}=v_{0}\;\theta_{hop}$$

(2) $$\theta_{hop}=\frac{\tau_{c}}{\tau_{c}+\tau_{t}}=\;\exp\;\left(-\frac{\varphi_{t}}{k\;T}\right)$$

where *v* is migration velocity, *E* is electric field strength, *μ* is carrier mobility, *θ_nop_* is ratio of free carrier density to total carrier density are calculated, *τ_c_* is hopping time, *τ_t_* is trapping time, *T* is absolute temperature, *φ_t_* is barrier height, *k* is Boltzmann constant.

**Figure 3:**
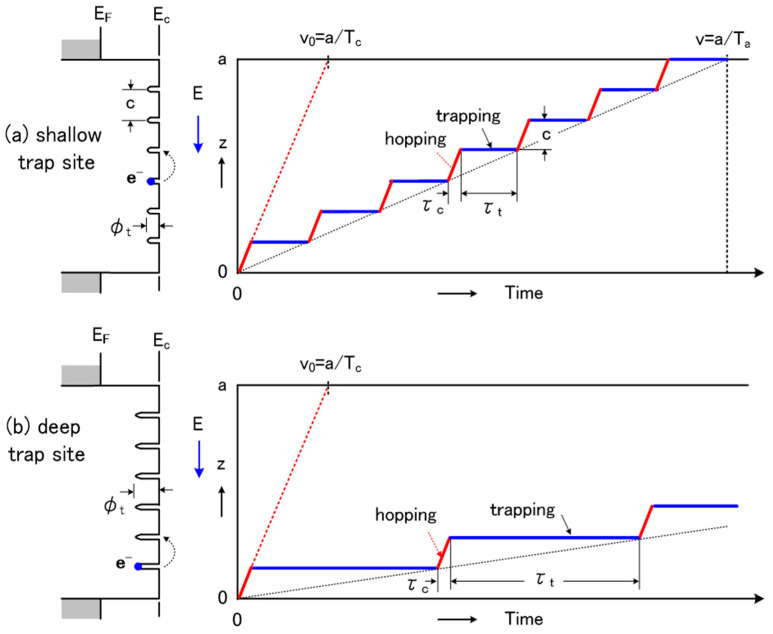
Relationship between carrier hopping process and trap sites. (**a**) Carrier hopping process for the shallow trap site; (**b**) Carrier hopping process for the deep trap site.

The relationship between the carrier hopping process and trap sites is shown in Figure 3. The carrier migration is determined by the trap distribution to a certain extent. Among shallow trap sites, the hopping probability of carriers is higher and migration time is shorter, which is due to the low barrier height, compared with that of deep trap sites. The analysis above can explain the intrinsic association among the trap sites, carrier hopping process and surface potential decay process. After fluorination, the traps become shallower; thus, the free carriers increase and the carriers obtain faster migration velocity, finally resulting in the dissipation of the surface charges.

### 3.2. Polarity Effects

**Figure 4:**
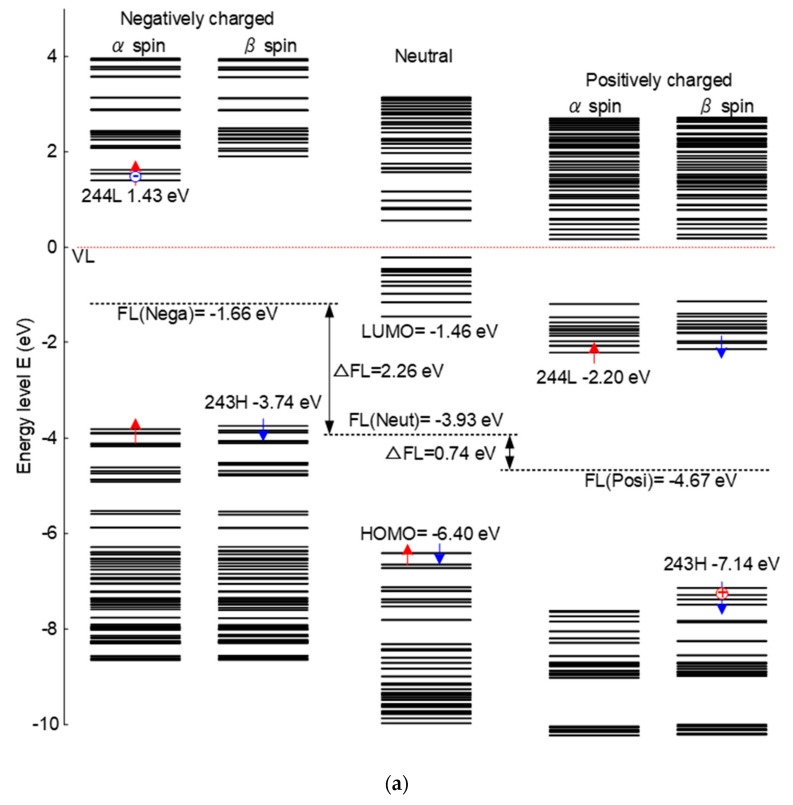

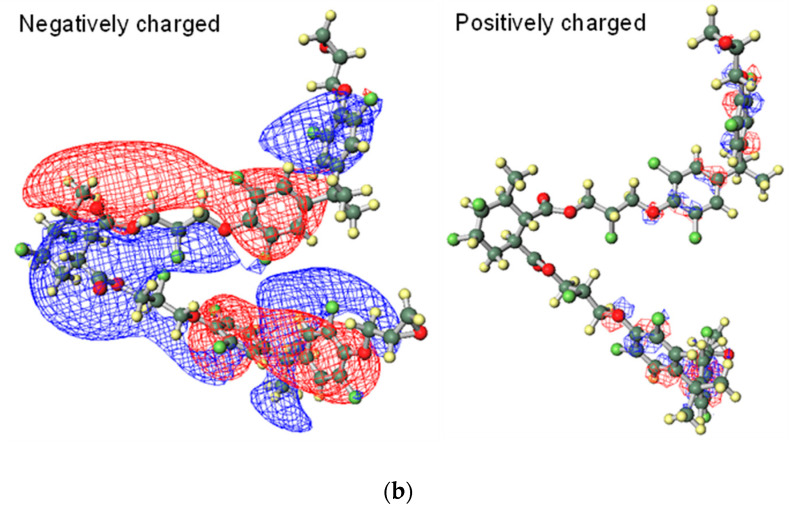
Energy level distribution and molecular orbitals of epoxy resin under different charging conditions. (**a**) Energy level distribution of different charged epoxy; (**b**) molecular orbitals of LUMO under negatively charging and HOMO under positively charging.

Figure 4 shows the energy level distribution and molecular orbitals of epoxy resin under different charging conditions. As is shown in Figure 4a, there is a significant change in energy level of positively or negatively charged epoxy. For the negatively charged condition, newly introduced energy levels, like 1.43 eV, show the state of trapped electrons. Additionally, there appears a new energy level at about −7.14 eV due to the effect of positive charges distributed in the epoxy samples. The whole distribution of the energy level is elevated when negatively charged and decreased when positive charged compared with the neutral situation. The distribution of energy levels determines the transition and transport of electrons.

Figure 4b shows the molecular orbitals of LUMO level under negatively charging and HOMO level under positively charging, which means the epoxy network is charged with different polarities. The red and blue parts represent different spin directions. For the negatively charging, it can be seen that electrons are distributed all along the epoxy molecular chains, which are relatively easy to migrate. However, as for the positively charged situation, electrons are limited in the local trap sites and are difficult to move freely. Thus, the macro electrical properties show a significant relationship with the structure of the molecule. The movement of the electrons are restricted based on the molecular orbitals of HOMO level under positively charging, correspondingly, the surface charge accumulation will be more serious under positive DC voltage.

## 4. Materials and Methods

### 4.1. Sample Preparation

First, sheet samples with a thickness of 0.05 cm and side length of 9 cm were obtained by hot pressing. The samples consist of epoxy matrix of CT 5531 and curing agent of HY 5533 from ARADUR^®^ (Shanghai, China). The fluorination process was conducted in a sealed reactor and the samples were treated with the F_2_/N_2_ gas mixture containing 20 vol% F_2_ under the temperature of 25 °C for 30 min. Based on our previous research, the fluorination time was chosen as 30 min to make a comparison with the original sample [2,18]. The surface charge decay process was tested with the temperature as a constant of 25 °C and the humidity kept as 28%, avoiding the error caused by the experimental environment and increasing the testing accuracy. The experimental setup and trap distribution calculation method were introduced in our previous paper [18].

### 4.2. QCC Calculation Model

**Figure 5:**
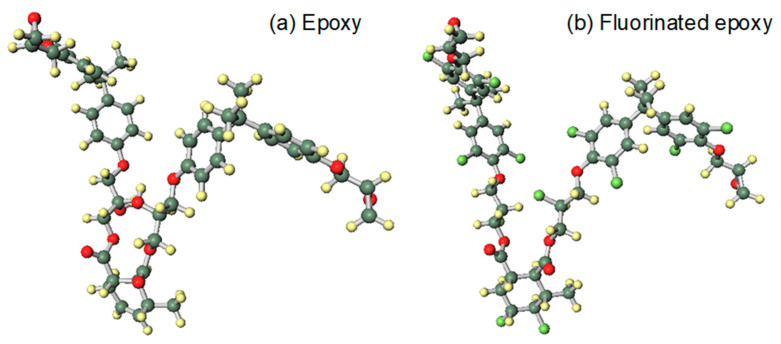
QCC models of epoxy resin before and after fluorination. (**a**) Model of epoxy resin; (**b**) Model of fluorinated epoxy resin.

The calculated structure is simplified from methyl-tetrahydrophthalic-anhydride cured bisphenol A epoxy resin. After fluorination, ortho hydrogen atoms are replaced by fluorine atoms and the double bond has an addition reaction, as shown in Figure 5. In this work, the kinetic energy distribution and molecular orbitals were calculated using DFT with the B3LYP hamiltonian and the 6-31G basis function in Gaussian 09 software (Wallingford, CT, USA). The calculation method was described in our previous paper [22,34].

## 5. Conclusions

In this paper, based on the experimental tests and the model analysis, the charging modulation mechanisms of epoxy resin were analyzed by quantum chemical calculation from the perspective of molecular orbital, and the relationship between the molecule structure and the electron migration is established. The conclusions can be summarized as follows.

After the corona charging treatment, the initial charge density of the fluorinated epoxy sample was reduced, and the rate of the surface charge dissipation was accelerated. Correspondingly, the density and the level of traps become lower compared with those of the original sample, affecting the carrier hopping process. The charges migration takes less time and holds a higher carrier mobility. Additionally, the positively or negatively charged epoxy possesses different molecular orbitals. Electrons tend to be distributed along the epoxy chain under negatively charging, which shows a greater probability of movement and is consistent with the law of surface charge dissipation.
